# Computing Highly Correlated Positions Using Mutual Information and Graph Theory for G Protein-Coupled Receptors

**DOI:** 10.1371/journal.pone.0004681

**Published:** 2009-03-05

**Authors:** Sarosh N. Fatakia, Stefano Costanzi, Carson C. Chow

**Affiliations:** Laboratory of Biological Modeling, National Institute of Diabetes and Digestive and Kidney Diseases, National Institutes of Health, Bethesda, Maryland, United States of America; Center for Genomic Regulation, Spain

## Abstract

G protein-coupled receptors (GPCRs) are a superfamily of seven transmembrane-spanning proteins involved in a wide array of physiological functions and are the most common targets of pharmaceuticals. This study aims to identify a cohort or clique of positions that share high mutual information. Using a multiple sequence alignment of the transmembrane (TM) domains, we calculated the mutual information between all inter-TM pairs of aligned positions and ranked the pairs by mutual information. A mutual information graph was constructed with vertices that corresponded to TM positions and edges between vertices were drawn if the mutual information exceeded a threshold of statistical significance. Positions with high degree (i.e. had significant mutual information with a large number of other positions) were found to line a well defined inter-TM ligand binding cavity for class A as well as class C GPCRs. Although the natural ligands of class C receptors bind to their extracellular N-terminal domains, the possibility of modulating their activity through ligands that bind to their helical bundle has been reported. Such positions were not found for class B GPCRs, in agreement with the observation that there are not known ligands that bind within their TM helical bundle. All identified key positions formed a clique within the MI graph of interest. For a subset of class A receptors we also considered the alignment of a portion of the second extracellular loop, and found that the two positions adjacent to the conserved Cys that bridges the loop with the TM3 qualified as key positions. Our algorithm may be useful for localizing topologically conserved regions in other protein families.

## Introduction

G protein-coupled receptors (GPCRs), with an estimated 1000 members [1], are the largest superfamily of membrane proteins in the human genome. They are critical for numerous vital cellular functions and their signaling governs various physiological and pathological processes. For these reasons, GPCRs are the most common targets for pharmacological intervention [2]. This study aims to identify a cohort or clique of non-conserved, yet correlated positions in GPCRs, common to the superfamily.

On the basis of whole sequence comparison, Fredriksson and coworkers classified human GPCRs into five distinct subfamilies: the rhodopsin family (R, also known as class A), the adhesion and secretin families (A and S, also known as class B), the glutamate family (G, also known as class C), and the frizzled/taste family (F, also known as class F) [3]. Structurally, GPCRs consist of a single polypeptide chain that crosses the plasma membrane seven times, with seven alpha-helical transmembrane domains (7-TMs) connected by three intracellular and three extracellular loops. The N-terminus is exterior to the cell, while the C-terminus is within the cytoplasm [4].

GPCR crystal structures, available for rhodopsin and two subtypes of the β-adrenergic receptors (β-ARs), and computational models supported by biochemical and molecular pharmacological data suggest the presence of a common binding cavity, located within the TMs toward the extracellular side of the helical bundle (7-TM cavity), considered to house the orthosteric ligand binding site for most receptors [5]–[15]. The recent publication of the crystal structure of the Adenosine A_2A_ receptor also supports the presence of a common binding cavity [16].

It is hypothesized that through gene duplications and subsequent mutations, common ancestor proteins gave rise to families of homologous proteins [17]. For paralogous protein superfamilies, such as the GPCR superfamily undertaken in our study, the germ of the function of novel proteins is usually present in its ancestor(s), and new proteins with novel functions arise mainly by the modulation of existing ones. In the course of this evolutionary process, some of the amino acid residues involved in the structure or function of proteins remained relatively conserved. Mutations at other positions, possibly followed by subsequent mutations elsewhere in the protein either preserved (or restored) the original protein function or gave rise to a newly acquired one. In this context, the identification of correlated residue positions in multi-sequence alignments (MSAs) can help to identify biologically relevant sets of residues and the functional surfaces that they form in protein superfamilies. For instance, in previous studies involving GPCRs, Oliveira and coworkers identified networks of correlated mutations consisting of positions involved in ligand binding, G protein coupling, and activation [18], while IJzerman and coworkers carried out an independent two-entropy analysis to determine the potential function of TM positions [19]. In the absence of comprehensive structural information from the entire GPCR superfamily, bioinformatic algorithms (such as those just mentioned) are used to predict specificity determining positions or functionally important positions solely from their sequences.

We propose a generic algorithm that identifies a cohort of correlated positions on the basis of mutual information and graph theory from any MSA of AA residues (this algorithm can be modified to incorporate nucleotide sequences). The algorithm does not incorporate any structural information involving positional specificity or physicochemical interactions amongst the residues involved. We focused on the 7-TMs from GPCRs because the MSA is devoid of gaps. Experimental evidence has also demonstrated that residues located in the second extracellular loop (EL2) constitute an integral part of the ligand-binding cavity of class A GPCRs and may play a role in receptor activation [5]–[7], [9], [20]–[28]. Thus, for a subset of class A, we added to the MSA of the 7-TMs the alignment of 5 contiguous EL2 residues.

**Figure 1:**
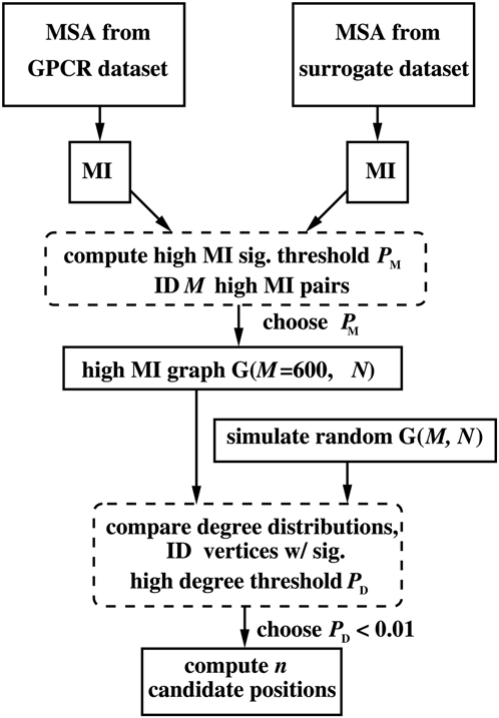
Algorithm. The flowchart of the algorithm used to establish candidate positions.

Our algorithm, described in a flowchart in Figure 1, involves the pre-selection of pairs of aligned positions on the basis of the mutual information (MI) between all possible inter-TM position pairs. The MI between two positions (or columns) within an MSA, represents the reduction in uncertainty of the residue at one position when the residue at the other position (for the corresponding sequence) is specified [29], [30]. The higher the MI value the greater the correlation or statistical dependence between the residues at the two positions. There exists a range of methods to identify correlated position pairs within an MSA using MI [31]–[57]. It has also been widely reported that positions sharing high MI with other positions are generally located within functionally important surfaces such as the ligand-binding sites and form a network or clique [31]–[38], [40]–[47], [49]–[55], [57]. For instance, from amongst the previously cited works, it has been specifically and independently reported that residues which exhibit correlated mutations in tandem with other residues are frequently located in protein active sites and binding interfaces [38], [51], [52].

However, to our knowledge there has not been a quantitative attempt to use concepts of graph theory to identify and characterize a densely or fully connected network (i.e. clique) of high MI position pairs in terms of a significantly high number of high MI connections (*degree*) each position shares with other positions. The novelty of our algorithm is in the extension of this MI approach by constructing abstract MI graphs, where positions were represented by vertices, and edges between vertices existed if the MI between that pair of vertices exceeded a significance threshold. The problem of clique identification in a graph is NP complete. To reduce the computational complexity, we focused on vertices in the graph that had a statistically significant degree, i.e. high connectivity with other vertices.

Our goal was to identify positions within the TMs that possess high MI with a large number of other positions on non-identical TMs. Given that MI can be influenced by random or phylogenetic sources, we also repeated our analysis with modified MI measures [37], [38], [50]. We found that for class A and C receptors, the vertices on the graph with high degree form a clique that correspond to positions located within the 7-TM cavity and line the experimentally determined or computationally proposed ligand binding sites, suggesting their coevolution and their ability of altering an essential component of the receptor function, i.e. ligand recognition. We also found that high degree vertices on the graph for class B receptors are not located within the 7-TM cavity in accordance with the fact that ligands that bind to their 7-TM helical bundle have not been identified. As mentioned, for a subset of class A receptors we also considered the alignment of a portion of the second extracellular loop (EL2), two residues of which qualified as key positions.

## Results

### Data set

We performed our analyses using a publicly available MSA relative to 7-TMs only, due to Rognan and colleagues (see Methods for details) [15]. The set contains 287, 49 and 22 sequences from classes A, B, and C GPCRs, respectively. This MSA primarily comprises receptors for which the natural ligand binding site is known or thought to be located within the 7-TM cavity, but also includes receptors the natural ligands of which bind to large N-terminal soluble ectodomains, such as the glycoprotein hormone receptor (GPHR) family of class A, and virtually all the members of class B and C GPCRs.

### Mutual Information

**Figure 2:**
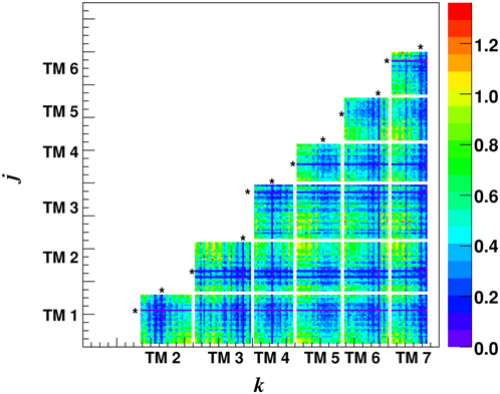
Mutual information values. Mutual information *MI(j,k)* values of all inter-TM position pairs for class A.

The MI for all inter-TM ordered pairs of positions was computed. To eliminate potential nearest neighbor interaction/correlation amongst residues within the same TMs, we did not include the intra-TM position pairs. We used MI as defined in Equation 1 of Methods section. This statistic is a function of the independent probabilities *p*(*x*) and *p*(*y*) for obtaining AA residues *x* and *y* at specific positions (in the MSA), as well as their joint probabilities *p*(*x,y*). The computed MI values are displayed on a 2D grid plot displaying the ordered pair of positions *(j,k)* on the vertical and horizontal axis respectively (only pairs with *j*<*k* are displayed). The inter-TM MI for class A receptors is shown in Figure 2. The asterisks mark the highly conserved positions named 1.50, 2.50, 3.50, 4.50, 5.50, 6.50 and 7.50 according to the Ballesteros and Weinstein [58] indexing scheme. The dark violet/blue striped patterns, corresponding to very low MI, demark the locations of highly conserved TM positions. For positions that are much more conserved, the joint probability between them and other less conserved positions is approximately equal to the product of the individual probabilities of the latter position, resulting in low MI (see Equation 1 in Methods). Nevertheless, such well conserved positions have been shown to be important in the structure and function of the receptors [4].

**Figure 3:**
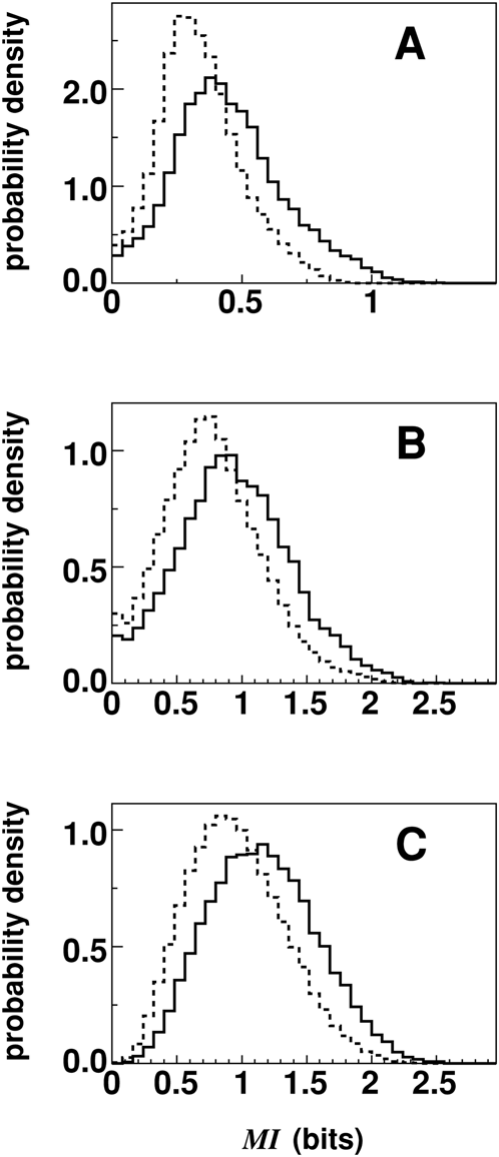
Probability density function. Probability density functions of *MI(j,k)* values for classes A, B and C (solid line) and for an ensemble of surrogate sets of random TMs (dotted line).

The probabilities used to compute the MI in Figure 2 were estimated from frequencies of AA appearances at each position or position pair. It is well known that estimating MI from a finite set of sequences will result in a finite-size error [56], [59]. As an example, for completely random sequences with complete statistical independence between positions, the theoretical MI between any two positions is zero because 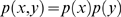. However, for a finite number of sequences *S*, it can be shown that the estimate for MI can be nonzero and scales as MI ∼log [1/*S*] (See Methods). Thus, to assess the significance of our estimated MI we compared it to a randomized/shuffled surrogate set with the same number of sequences, as described by Mirny and Gelfand [51]. By shuffling residues among the sequences at the same MSA alignment position, simulated surrogate sets that preserved the residue probabilities *p(x)* and *p(y)* but randomized the joint probabilities *p(x,y)* were obtained (i.e. the joint entropy was maximized by shuffling). As a null hypothesis, we attributed non-zero MI values to arise from finite-size errors as represented by the surrogate simulations. The alternate hypothesis was that pairs of positions with high MI values represented true correlations. These correlations could possibly be due to coevolving residues, correlated mutations, phylogenetic noise, a biased dataset, or a combination of these factors [32]. The probability density function (PDF) representing the MI values for classes A, B, and C along with the surrogate set of randomized sequences is shown in Figure 3. The figures show that the PDFs are highly skewed and finite-size errors can be quite large (as evidenced by the surrogate set PDFs) especially for the smaller datasets.

### MI Graph

We constructed a MI graph with *N* vertices that represented TM positions and the *M* edges that represented highly significant MI values with respect to the surrogate set. Given that MI is a pair-wise measure, we used two elementary concepts from graph theory to construct the graph and uncover a network of correlated positions. We used *closeness centrality* to pre-select position pairs to define the edges of a MI graph and *degree centrality* to identify highly connected positions in the MI graph. We utilize two elementary graph theoretic measures to analyze MI graphs. The MI values that were significantly larger than those of the random surrogate set of sequences were selected to construct the MI graph. Since only edges with high MI were included, the resulting MI graph is not complete (i.e. every vertex is not connected to every other vertex). A range of P values for assigning the significance level, denoted by *P*_M_, resulted in different sized graphs. For classes B and C, varying *P*_M_ (given by 0.010<*P*_M_<0.015) resulted in MI graphs with the number of edges *M* ranging from 300 to 700. For class A, the same range of *M* was obtained for *P*_M_ that was an order of magnitude smaller as evident in Figure 3. Since a given value of *P*_M_ corresponds to a unique *M*, we use both values interchangeably when describing the MI graph.

**Figure 4:**
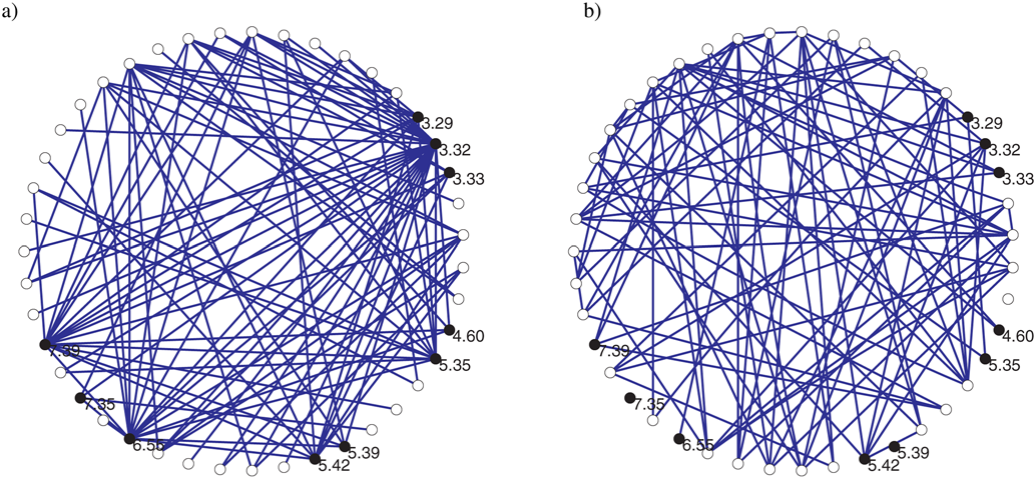
Mutual information graphs. (a) Class A MI graph for 100 edges with the highest MI. Solid black vertices are the 10 key positions. (b) Example random graph with 100 edges.

**Figure 5:**
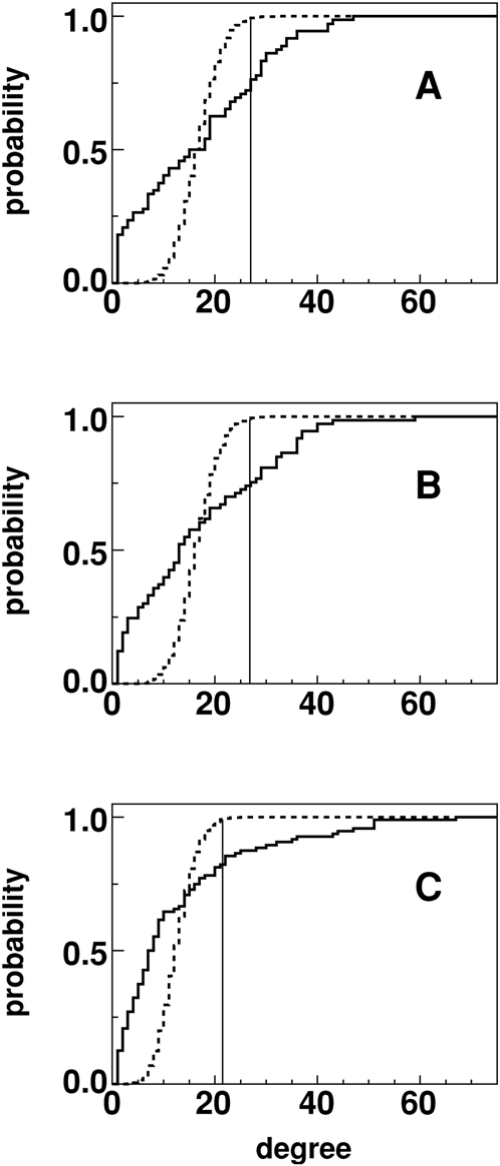
Degree distributions. Cumulative degree distribution function for class A, B and C MI graph (solid line) and simulated random graphs (dotted line). Degree values higher than the vertical line have *P*_D_<0.010.

A representative MI graph for class A with 100 edges is shown in Figure 4a. Here vertices of the MI graph are arranged on a circular ring in the order of the corresponding position location on the 7-TMs. Lines connecting the vertices represent edges indicating significant MI between the position pairs. From the graph, one can clearly see that some vertices have many more edges than other vertices (i.e. higher degree) when contrasted with a graph having identical *M* and *N* but obtained via random connections (Figure 4b). In Figure 5, the distribution for the number of vertices with given degree for the MI graphs (using *M* = 600) and a set of random graphs having identical number of vertices and edges is shown. The vertical line corresponds to *P*_D_<0.010, (the choice of which is motivated later) implying that vertices with degree exceeding 27, 27 and 22 edges for classes A, B and C respectively are significant to this P value.

### Key positions

We hypothesized that the non-conserved correlated positions, called key positions, corresponded to vertices with high *degree* (i.e. have a large number of edges incident to them) and identified the significant high degree positions for the three classes of GPCRs. Statistical significance was measured in terms of a P value (*P*_D_) with respect to a simulated set of over twenty thousand random graphs with identical *M* and *N*. Since the degree distribution evolved by changing *M*, a different choice of *P*_M_ and *P*_D_ resulted in a different cohort of high degree vertices, which we called candidate positions.

To decide upon a single clique or cohort of key positions, we used an additional criterion of invariance to changes in *P*_M_, *P*_D_ and a leave-one-out analysis for sequences. For a range of M values (50≤*M*≤2050 in steps of 50), we found that for *P*_D_<0.010 the candidate positions were mostly invariant to the leave-one-out analysis depending on *P*_M_. As *M* increased, the number of candidate positions also increased but not all of the candidate positions were invariant (i.e. positions found for a lower value of *M* were not necessarily found for a higher value of *M*). For *M*<500, none of the three classes had an invariant cohort. For class A, an invariant cohort existed for *M* = 500, 550, 600 and 750 (and none for *M* = 650, 700, or from *M* = 800 up to *M* = 1400). The minimum value of *M* for which a stable and invariant cohort of candidate positions consistently appeared in classes A, B and C was *M* = 600 and these positions were selected as the cohort of key positions. Rank-wise, these pairs were among the top 4% of the inter-TM MI pairs. Using *P*_D_ = 0.010 and *M* = 600, resulted in 10 key positions for class A and 9 positions for classes B and C, which are listed in Table 1. All the key positions were connected to all the other key positions (within the graph of interest) so the key positions obtained from all three classes form a clique.

**Table 1:** Identified key positions (Ballesteros-Weinstein index) for class A (exclusively involving the 7-TMs as well as the EL2) and for the 7-TMs from classes B and C.

	class A	class A w/EL2	class B	class C
**TM1**				1.33
				1.36
**TM2**			2.38	
			2.54	
			2.62	
		2.67		
**TM3**			3.22	
				3.26
	3.29	3.29		
	3.32	3.32		
	3.33	3.33		
**TM4**			4.41	
	4.60	4.60		
				4.61
**EL2**		EL2.49		
		EL2.51		
**TM5**	5.35	5.35		
			5.38	
	5.39	5.39		
	5.42	5.42		5.42
				5.43
			5.44	
**TM6**			6.30	
			6.33	
	6.55	6.55		
**TM7**	7.35	7.35		
				7.36
	7.39	7.39		7.39
				7.40

**Figure 6:**
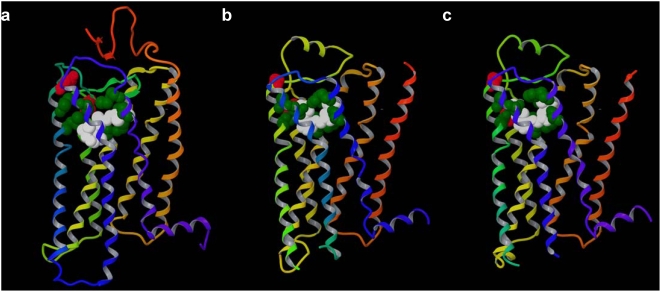
Class A key positions. Class A key positions visualized in the crystal structure of rhodopsin (a), β_2_-AR (b), and β_1_-AR (c) respecitvely. All the key positions are located in the exofacial 7-TM binding cavity. Residues at positions 4.60 and 5.35 (in red) can be considered hinges for EL2. All the residues at the remaining key positions (in green) directly line the cavities (pockets) for the co-crystallized ligands (in white). Ligands and residues at key positions are represented as space filling models. The backbone of the receptor is schematically represented as a ribbon, depicted with the colors of the rainbow from the N-terminus to the C-terminus (TM1: red; TM2: orange; TM3: yellow; TM4: yellow/green; TM5: green; TM6: cyan; TM7: blue).

The key positions of class A GPCRs, calculated on the basis of the alignment of the 7-TMs, are visualized in Figure 6 in the 3D crystallographic structures of rhodopsin (panel a), the β_2_-AR (panel b) and the β_1_-AR (panel c). Table 2 reports all residues in the 7-TMs of rhodopsin, the β_2_-AR and the adenosine A_2A_ receptor that are in contact with the co-crystallized ligand (underlined entries) and all the residues predicted in this study as key positions (denoted in bold and marked with an X). The table also reports the MI data relative to the listed residues. As evident from Figure 6 and Table 2, the cohort of key positions resulting from the analysis of class A receptors consists of residues that are all located in the exofacial 7-TM-binding cavity and that, with two notable exceptions (i.e. positions 4.60 and 5.35), closely surround the synthetic inverse agonists and antagonists co-crystallized with the β-ARs and the adenosine A_2A_ receptor, and the natural inverse agonist 11-*cis*-retinal covalently bound to rhodopsin. In particular, with the exceptions of P4.60 and N5.35, all of the residues at the key positions establish direct contacts with carazolol in the β_2_-AR. The key positions 4.60 and 5.35 are located at the C-terminal end of TM4 and the N-terminal end of TM5, respectively, and can be regarded as two hinges connecting EL2 with TM4 and TM5. We argue that the biological significance of these residues could be linked to their role in the proposed functionally relevant ligand-induced conformational changes of EL2 leading to receptor activation, rather than to interactions with ligands [21], [28]. In the crystal structure of rhodopsin, residues at three additional key positions, namely V5.39, A6.55, and M7.35, are not in direct contact with the ligand. However, these residues are located in proximity to retinal and are in direct contact with three (in the case of M7.35) or four (in the case of V5.39 and A6.55) residues that establish contacts with it, and thus can be considered an integral part of the binding cavity. In the crystal structure of the A_2A_ receptor, besides the two hinges of EL2, there are two additional key positions that are not in direct contact with the ligand, namely, A3.28 and V5.39. Also in this case, these residues are in direct contact with residues that, in turn, establish fundamental interactions with the ligand, namely F(EL2.52) and N(6.55). These data demonstrate that our key positions identify very well the binding pockets of all four crystallized receptors.

**Table 2:** List of positions located in the 7-TMs of rhodopsin (Rho), beta-2 adrenergic receptor (β_2_-AR) and adenosine receptor (A_2A_) that are in contact with the co-crystallized ligand (underlined) and/or qualified as key positions (in bold and marked with an ***X***).

position	Rho	β_2_-AR	A_2A_	key	degree	entropy	Pair position (pp)		
							Pp	rank	MI
3.28	Glu 113	Trp 109	Ile 80		29	3.742	5.35	60	1.053
**3.29**	***Gly 114***	***Thr 110***	**Ala81**	***X***	32	3.883	5.35	14	1.153
**3.32**	***Ala 117***	***Asp 113***	***Val 84***	***X***	52	3.724	2.57	2	1.287
**3.33**	***Thr 118***	***Val 114***	***Lue 85***	***X***	37	3.772	6.55	9	1.167
3.36	Gly 121	Val 117	Thr 88		31	3.522	2.57	8	1.173
3.37	Glu 122	Thr 118	Gln 89		18	3.269	6.52	24	1.107
**4.60**	**Pro 171**	**Pro 168**	**Pro139**	***X***	39	3.604	7.39	7	1.191
**5.35**	**Asn 200**	**Asn 196**	**Met 174**	***X***	40	4.106	3.32	10	1.167
5.38	Phe 203	Tyr 199	Met 177		10	3.580	7.35	59	1.054
**5.39**	**Val 204**	***Ala 200***	**Val 178**	***X***	40	3.853	2.64	23	1.114
**5.42**	***Met207***	***Ser 203***	***Asn 181***	***X***	39	3.848	3.29	25	1.107
5.43	Phe208	Ser 204	Phe 182		11	3.507	3.32	62	1.051
5.46	His211	Ser 207	Cys 185		32	3.470	3.32	13	1.162
5.47	Phe212	Phe 208	Val 186		n.a.	-	3.32	1013	0.801
6.44	Phe261	Phe 282	Phe 242		n.a.	1.275	7.43	7472	0.435
6.48	Trp265	Trp 286	Trp 246		n.a.	1.707	2.60	1453	0.749
6.51	Tyr268	Phe 289	Leu 249		n.a.	2.577	3.32	901	0.817
6.52	Ala269	Phe 290	His 250		16	3.333	3.37	24	1.107
**6.55**	**Ala272**	***Asn 293***	***Asn 253***	***X***	45	3.976	7.39	1	1.362
**7.35**	**Met288**	***Tyr 308***	***Met 270***	***X***	39	3.794	2.64	33	1.097
**7.39**	***Ala292***	***Asn 312***	***Ile 274***	***X***	49	3.806	6.55	1	1.362
7.42	Ala295	Gly315	Ser 277		1	2.753	3.32	712	0.851
7.43	Lys296	Tyr 316	His 278		15	3.293	3.32	5	1.232

The MI data refer to the analysis performed on the MSA of the 7-TMs class A receptors. The position has highest MI (among all other inter-TM positions) with the listed pair position (pp). Rank is the MI ranking of that pair using the class A 7-TM MSA (287 sequences) and the degree is calculated using MI graph from top 600 MI pairs. Positions not included in the MI graph have “n.a.” ascribed to the degree value. Entropy and MI are measured in bits.

Among the key positions indicated in Figure 6, particularly important in the β_2_-AR-carazolol interactions are positions D3.32 and N7.39, which coordinate the positively charged amino group of carazolol, and S5.42, which coordinates its aromatic amine. These residues are maintained to establish fundamental interactions also with the natural agonists epinephrine and norepinephrine [60]–[62]. In rhodopsin, the side chain of positions G3.29, A3.32, T3.33, and A7.39 surround the polyene chain of retinal, with T3.33 contributing to the position of the C9-methyl group, while M5.42 interacts with the β-ionone ring [5].

Residues located at the key positions identified in this work, have been experimentally demonstrated to be implicated in ligand recognition in several systems, including, among many others, adenosine, serotonin, P2Y, and free fatty acid receptors [20], [28], [63]–[66]. Our analysis also included the sequences of the class A receptors which are naturally activated by large peptides that bind to their N-termini, such as glycoprotein hormone receptors (GPHRs). Mutagenesis data and chemical modification of the ligands demonstrated that, as supported by our analysis, also the activity of these receptors can be modulated through synthetic low molecular weight compounds that allosterically interact with the 7-TM binding cavity [67]–[69].

**Figure 7:**
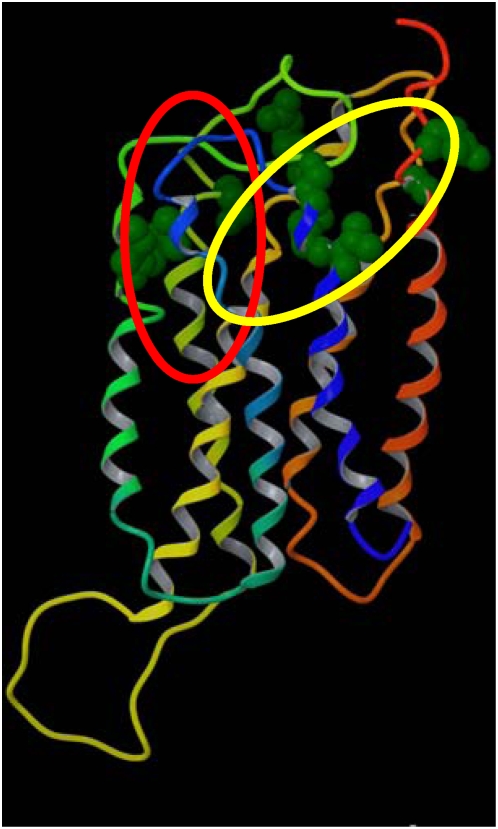
Class C key positions. Class C key positions visualized in a molecular model of the Ca^2+^ receptors. The key positions (in green) are located in correspondence with two predicted adjacent sites for different classes of allosteric modulators that bind to the exofacial 7-TM cavity of the receptor [58]. Although the natural ligands of class C receptors bind to their large N-terminal ectodomain, our analysis supports experimental evidence that their activity can be modulated through molecules that allosterically bind within the transmembrane helical bundle. Ligands and residues at key positions are represented as space filling models. The backbone of the receptor is schematically represented as a ribbon, depicted with the colors of the rainbow from the N-terminus to the C-terminus (TM1: red; TM2: orange; TM3: yellow; TM4: yellow/green; TM5: green; TM6: cyan; TM7: blue).

It was also found that for class C receptors the key positions were located within the exofacial 7-TM cavity, in proximity to the orthosteric binding site crystallographically identified for rhodopsin and the β-ARs (Figure 7). Although the natural ligands of class C receptors bind to their N-terminal domains, a number of articles, in agreement with our results, have reported the possibility of allosterically modulating their activity through ligands that bind to the 7-TM cavity [70]–[76]. While for class A receptors the key positions are concentrated in a region between TM3, TM5, TM6, and TM7, for class C receptors they are more widely spread out throughout the whole upper portion of the helical bundle, encompassing residues from TM1 too. Molecular modeling and mutagenesis data have consistently suggested the presence of two adjacent potential sites of binding for different classes of allosteric modulators of the human Ca^2+^ receptor – a member of class C – located within the upper part of the helical bundle. As shown in Figure 7, all the identified key positions fall within the two adjacent sites proposed in the published *in silico* model [71].

**Figure 8:**
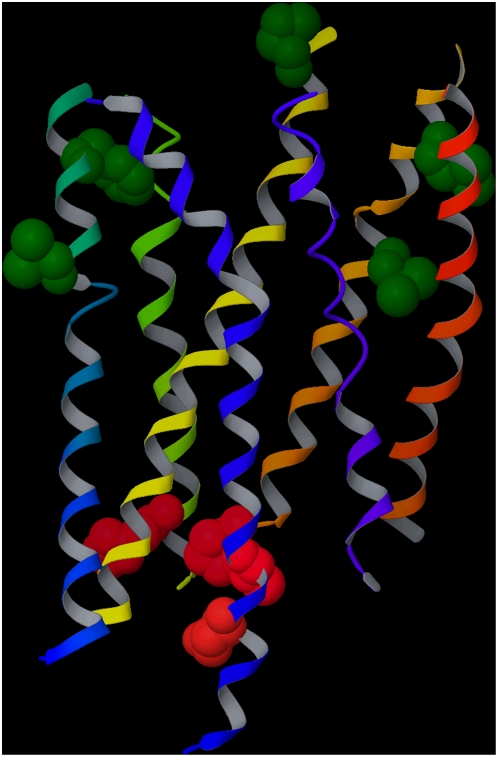
Class B key positions. Class B key positions visualized in the crystal structure of rhodopsin (a class A receptor). Unlike in the case of class A and class C, the key positions identified for class B receptors are localized in two different areas: five of them (in green) are located within the exofacial 7-TM cavity, while the remaining four (in red) are located near the intracellular loop. Ligands and residues at key positions are represented as space filling models. The backbone of the receptor is schematically represented as a ribbon, depicted with the colors of the rainbow from the N-terminus to the C-terminus (TM1: red; TM2: orange; TM3: yellow; TM4: yellow/green; TM5: green; TM6: cyan; TM7: blue). Although the topology of the TM helices appears to be substantially conserved within class A GPCRs, the topology of family B receptors may be different from that of rhodopsin, thus the figure is only intended as a schematic. We do not represent the extra and intracellular portions of the receptor, since they are not conserved.

The key positions identified from the analysis of class B receptors, whose natural ligands also bind extracellularly through the N-terminal domain, are not located in a common binding cavity but concentrated in two regions (Figure 8). Five of them – namely 2.54, 2.62, 3.22, 5.38, 5.44 – are located toward the extracellular side of the helical bundle, loosely in correspondence of the 7-TM binding cavity. However, the remaining four – namely 2.38, 4.41, 6.30, and 6.33 – are located near the intracellular loops. These key positions were not identified in a topologically ordered manner. To the best of our knowledge, ligands that bind to the 7-TM cavity of class B receptors have not been found. Hence, the lack of a contiguous cohort of high degree positions is consistent with the absence of a clearly defined 7-TM binding cavity for class B receptors.

### Second extracellular loop (EL2)

Given that the portion of EL2 connected via a disulphide bridge to TM3 has been shown to be involved in ligand recognition and receptor activation for a number of class A GPCRs [21], [23]–[28], we also investigated if any of the EL2 positions could be identified. Thus, for a subset composed of 249 class A receptors, we added to the MSA of the 7-TMs the alignment of 5 contiguous EL2 residues, starting at the position immediately preceding the conserved Cys residue (whose position is identified here as EL2.50) involved in the disulfide bridge with a second conserved Cys located at the extracellular end of TM3 (position 3.25, according to the Ballesteros and Weinstein scheme [58]). The alignment is provided as supporting info. The receptors missing either the Cys at position EL2.50 or the Cys at position 3.25, hence lacking the disulfide bridge, were excluded from this additional analysis.

For the analysis involving the EL2 region, the consistently invariant cohort was obtained up to *M* = 700 (unlike the previous case which involved the 7-TMs, for which the invariance was limited to *M* = 600). Hence, the largest invariant cohort of key positions was obtained for *M* = 700 from which 13 key positions were identified (Table 1). All the key positions formed a clique in the independent analysis. Amongst them were the 10 previously identified key class A positions and one new TM position (2.67). The other two positions, EL2.49 and EL2.51, were from EL2. Notably, these two positions are the nearest neighbors of the conserved Cys at position EL2.50 and were among the top 10 high degree positions (out of the set of 13) in the MI graph of interest. Notably residues at position EL2.51 are in contact with the co-crystallized ligand in rhodopsin and the A_2A_ receptor, but not in the β-ARs. Position EL2.52 forms extensive contacts with the co-crystallized ligand in the β_2_-AR and the A_2A_ receptor [5], [12], and has been proposed to be functionally important also for other GPCRs on the basis of molecular modeling. This position shares significantly high MI with other positions, but it does not have high enough connectivity with other TM residues to qualify as a key position. Table 3 is analogous to Table 2, but, in addition to the residues in the 7-TMs, it also reports those residues located in EL2 of rhodopsin, the β_2_-AR and the adenosine A_2A_ receptor that are in contact with the co-crystallized ligands (underlined entries) and/or are predicted in this study as key positions (denoted in bold and marked with an X). The MI data reported in the Table refer to the independent analysis performed while including the five EL2 positions.

**Table 3 pone-0004681-t003:** List of positions located in the 7-TMs and EL2 of rhodopsin (Rho).

Position	Rho	β_2_-AR	A_2A_	key	degree	entropy	Pair position (pp)		
							pp	rank	MI
**2.67**	**His 100**	**Met 96**	**Gly 69**	***X***	45	4.021	7.39	25	1.164
3.28	Glu 113	Trp 109	Ile 80		30	3.650	7.39	107	1.073
**3.29**	***Gly 114***	***Thr 110***	**Ala81**	***X***	35	3.743	5.35	18	1.189
**3.32**	***Ala 117***	***Asp 113***	***Val 84***	***X***	58	3.665	7.39	2	1.371
**3.33**	***Thr 118***	***Val 114***	***Lue 85***	***X***	41	3.733	6.55	21	1.176
3.36	Gly 121	Val 117	Thr 88		34	3.474	7.39	15	1.208
3.37	Glu 122	Thr 118	Gln 89		20	3.193	6.52	13	1.214
**4.60**	**Pro 171**	**Pro 168**	**Pro139**	***X***	36	3.645	7.39	9	1.249
EL2.49	**Ser 186**	**Cys 190**	**Ala 165**	**X**	47	4.051	5.35	34	1.142
EL2.50	Cys 187	Cys 191	*Cys 166*		n.a.	-	-	-	0.0
EL2.51	**Gly 188**	**Asp 192**	**Leu 167**	**X**	33	3.949	6.55	75	1.094
EL2.52	Ile 189	Phe 193	Phe 168		22	3.737	3.32	14	1.210
EL2.54	Tyr 191	Thr 195	Asp 170		-	-	-	-	-
**5.35**	**Asn 200**	**Asn 196**	**Met 174**	***X***	45	4.118	7.39	7	1.261
5.38	Phe 203	Tyr 199	Met 177		10	3.383	7.35	91	1.084
**5.39**	**Val 204**	***Ala 200***	**Val 178**	***X***	41	3.849	2.64	20	1.187
**5.42**	***Met 207***	***Ser 203***	***Asn 181***	***X***	41	3.777	3.32	33	1.143
5.43	Phe 208	Ser 204	Phe 182		11	3.440	3.32	114	1.068
5.46	His 211	Ser 207	Cys 185		31	3.423	3.32	4	1.293
5.47	Phe 212	Phe 208	Val 186		n.a.	-	3.32	866	0.874
6.44	Phe 261	Phe 282	Phe 242		n.a.	1.347	3.32	6029	0.523
6.48	Trp 265	Trp 286	Trp 246		n.a.	1.615	3.32	1755	0.764
6.51	Tyr 268	Phe 289	Leu 249		n.a.	2.410	3.32	1685	0.771
6.52	Ala 269	Phe 290	His 250		14	3.195	3.37	13	1.214
**6.55**	**Ala 272**	***Asn 293***	***Asn 253***	***X***	37	3.889	7.39	1	1.391
**7.35**	**Met 288**	***Tyr 308***	***Met 270***	***X***	31	3.755	3.32	23	1.166
**7.39**	***Ala 292***	***Asn 312***	***Ile 274***	***X***	47	3.815	6.55	1	1.391
7.42	Ala 295	Gly 315	Ser 277		n.a.	2.736	3.32	696	0.902
7.43	Lys 296	Tyr 316	His 278		8	3.099	3.32	5	1.282

beta-2 adrenergic receptor (β_2_-AR) and adenosine receptor (A_2A_) that are in contact with the co-crystallized ligand (underlined) and/or qualified as key positions (in bold and marked with an ***X***). The MI data refer to the analysis performed on a subset of class A receptor adding to the MSA of the 7-TMs the alignment of five EL2 positions. The position has highest MI (among all other inter-TM positions) with the listed pair position (pp). Rank is the MI ranking of that pair using a subset of class A 7-TM along with EL2 MSA (249 sequences) the degree is calculated using MI graph from top 700 MI pairs. Entropy and MI are measured in bits.

### Additional tests

**Figure 9:**
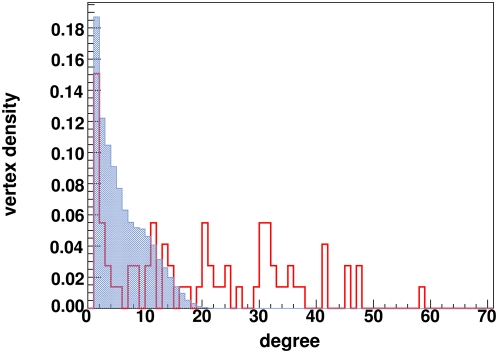
Comparison of degree distributions. The degree distribution from *M* = 700 MI graphs obtained from MI involving the 7TMs from class A dataset as well as the EL2 in red (dark). For comparison, the degree distribution from surrogate subsets (light blue) is overlaid.

In addition to testing the significance of our degree distribution against one generated from a set of random graphs, we also tested against the degree distribution of graphs generated directly from the surrogate set of shuffled sequences (null hypothesis). We again found that there existed vertices in the MI graph that had degree that were (statistically) significantly higher than the surrogate graphs. The degree distribution of the dataset involving receptors from class A along with EL2 is shown in Figure 9. The open (red) histogram represents the degree from the dataset and the filled (blue) histogram corresponds to the degree distribution obtained from the surrogate sets. The key positions are clearly statistically significant with respect to the surrogate degree distribution. However, estimates from classes B and C have yielded ∼20% and ∼15% chances for key positions to arise from the null hypothesis respectively (results not shown), indicating the possibility for false positive identification. Along these lines, we note that while both classes B and C did have 10 candidate positions, only 9 of them were invariant and consistently appeared in the top 10 ranks from the full dataset and all leave-one-out studies.

We also performed analyses similar to that proposed by Gloor et al. [38], [50], which is summarized in the Methods section. Their selection criteria were based on high normalized MI position pairs (MI normalized by the joint information entropy of residues at the paired positions) with Z-score>4.0, which is a threshold analogous to *P*_M_. They then selected positions that shared high normalized MI with three or more positions (i.e. degree≥3), which as analogous to our degree threshold *P*_D_. There were 20 TM positions that shared high normalized MI with other positions and 13 of those had a degree≥3. Four of those 13 positions (3.29, 5.35, 6.55 and 7.39) were common to those within the binding cavity listed in Table 2. In addition there were 2 positions (5.36 and 7.36) which happened to be nearest neighbors of positions in the binding cavity. The remaining 7 positions did not directly line the binding cavities of carazolol and retinal, but were located toward the extracellular side of the 7-TM binding cavity (data not shown).

When we repeated this analysis using un-normalized MI (defined in Equation 1), we obtained two pairs of positions that had Z-score>4.0. However, with a relaxed significance threshold of Z-score>3.0, we identified 9 positions having degree≥3. Seven of those 9 positions overlapped with our 10 key positions, while two of the positions were in exofacial side of TM 2 (2.57 and 2.64) in proximity, although not in direct contact with the ligands co-crystallized with rhodopsin and the β-ARs. For classes B and C, two and four positions, respectively, were obtained (data not shown) and these positions overlapped with our key positions. As an aside, we note that in our analysis for class A, the top 10 MI position pairs overlapped with only 6 of the 10 key TM positions, with the tenth key position ranked 33 in terms of MI. These results indicate that prioritizing selection of positions by high degree rather than by high MI may be more useful for identifying the ligand-binding cavity of GPCRs.

Dunn et al. [37] established a method to obtain a correction term to MI due to possible random or phylogenetic influences. We used this method to compute the corrected MI and repeated the analysis and obtained 11 key positions. These are compared to the known ligand binding positions in Table 4. Of the nine true positives, seven (3.29, 3.32, 3.33, 5.35, 5.42, 6.55 and 7.39) had been identified earlier using the raw MI. The two other positions which were not identified initially are 3.36 and 6.52. Two positions (2.57 and 2.67) are identified as false positives. A similar approach for class B and class C yielded eight positions that were common with the previously obtained nine key positions. For class B, position 1.31 was the new key position. For class C, 3.51 was the new position.

**Table 4:** List of leading positions located in the 7-TMs of the β_2_-AR and rhodopsin (Rho) that are in contact with the co-crystallized ligand (underlined) and/or qualified as leading positions (in bold and marked with an *).

Position	Rho	β_2_-AR	A_2A_	Key	Degree	entropy	Pair position (pp)		
							pp	rank	MIp
**2.57**	**Gly 90**	**Val 86**	**Ala 59**	*	35	3.214	3.32	6	1.068
**2.67**	**His 100**	**Met 96**	**Gly 69**	*	34	4.052	3.32	7	1.063
3.28	Glu 113	Trp 109	Ile 80		32	3.742	6.55	27	1.026
**3.29**	***Gly 114***	***Thr 110***	**Ala81**	*	38	3.883	6.55	12	1.050
**3.32**	***Ala 117***	***Asp 113***	***Val 84***	*	50	3.724	6.55	1	1.156
**3.33**	***Thr 118***	***Val 114***	***Lue 85***	*	34	3.772	6.55	24	1.030
**3.36**	Gly 121	Val 117	Thr 88	*	35	3.522	6.55	22	1.032
3.37	Glu 122	Thr 118	Gln 89		17	3.269	6.55	93	0.961
**5.35**	**Asn 200**	**Asn 196**	**Met 174**	*	38	4.106	3.32	5	1.076
5.38	Phe 203	Tyr 199	Met 177		18	3.580	3.32	67	0.980
5.39	**Val 204**	***Ala 200***	**Val 178**		31	3.853	3.32	15	1.043
**5.42**	***Met 207***	***Ser 203***	***Asn 181***	*	38	3.848	3.32	4	1.084
5.43	Phe 208	Ser 204	Phe 182		8	3.507	3.32	111	0.952
5.46	His 211	Ser 207	Cys 185		19	3.470	3.32	54	0.991
5.47	Phe 212	Phe 208	Val 186		n.a.	-	3.32	3259	0.604
6.44	Phe 261	Phe 282	Phe 242		n.a.	1.275	3.32	7329	0.444
6.48	Trp 265	Trp 286	Trp 246		n.a.	1.707	3.32	2461	0.654
6.51	Tyr 268	Phe 289	Leu 249		n.a.	2.577	3.32	1497	0.733
**6.52**	Ala 269	Phe 290	His 250	*	35	3.333	3.32	19	1.037
**6.55**	**Ala 272**	***Asn 293***	***Asn 253***	*	51	3.976	3.32	1	1.156
7.35	**Met 288**	***Tyr 308***	***Met 270***		32	3.794	3.32	13	1.047
**7.39**	***Ala 292***	***Asn 312***	***Ile 274***	*	45	3.806	3.32	2	1.120
7.42	Ala 295	Gly 315	Ser 277		n.a.	2.753	3.32	1190	0.767
7.43	Lys 296	Tyr 316	His 278		22	3.293	3.32	51	0.997

The MI data refer to the analysis performed on the MSA of the 7-TMs class A receptors using Dunn, Wahl and Gloor's (2008) method [37] to obtain MIp the corrected MI. The position has highest MI (among all other inter-TM positions) with the listed pair position (pp). Rank is the MI ranking of that pair using the class A 7-TM MSA (287 sequences) and the degree is calculated using MIp graph from top 600 MIp pairs. Positions are not included in the MIp graph have “n.a.” ascribed to the degree value. Entropy and MI are measured in bits.

### Sensitivity and specificity of key positions

**Figure 10:**
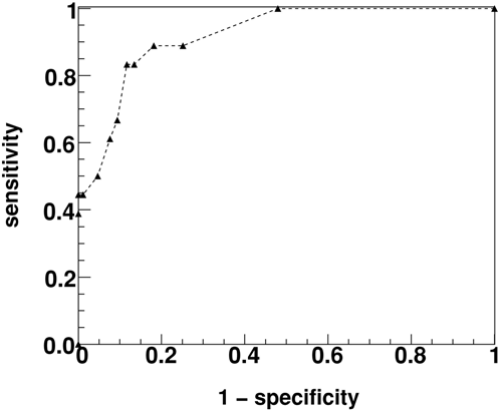
ROC curve. The measure of sensitivity versus (1- specificity) obtained for the beta2-adrenergic receptor 3D structure. The different points represent the varied significance threshold *P*_M_ for generating the MI graphs. Lower *P*_M_ values correspond to the region with the greatest specificity and moderate sensitivity.

We computed the sensitivity and specificity of our algorithm to predict the ligand binding-positions of the β_2_-AR structure. We computed the sensitivity and specificity for a range of threshold *P*_M_ values and plotted the ROC curve i.e. sensitivity versus (1 - specificity) (see Figure 10). For low *P*_M_ values the algorithm is highly specific in identifying the cohort of positions within the ligand-binding region. The sensitivity has a value near 0.5 for a specificity of 1. The estimated area under the ROC curve is 0.92, which indicates a high level of discrimination.

## Discussion

Using a novel algorithm based on mutual information and graph theory, we identified a clique of non-conserved positions in the 7-TM alignment of GPCRs that have a high degree of connectivity to other positions with respect to MI, i.e. high degree, high MI cohort. In addition, for a given statistical significance, the method provided a list of positions in hierarchical order of their connectivity. These key TM positions, which have been identified solely on the basis of sequence analysis without any prior hypotheses or knowledge involving the biological or structural role of specific residues, are all located within the 7-TM binding cavity for classes A and C but not for class B, where experimental evidence for a common binding cavity does not exist. The key positions also form a MI clique.

The visualization of the key positions for class A receptors in the crystal structures of the β-ARs and rhodopsin revealed that these positions closely surround the co-crystallized ligands (Figure 6). The finding has suggested that the 7-TM binding cavity might be even more topologically conserved than previously thought [12]–[15], and is crystallographically supported by the substantial overlap of retinal bound to rhodopsin and the synthetic inverse agonists cyanopindolol and carazolol bound to the β_1_- and β_2_-AR, respectively. Although the binding mode of ZM241385 – the synthetic antagonist co-crystallyzed with the A_2A_ receptor – shows some dissimilarities when compared to carazolol and retinal, the binding pocket identified by our key positions matches that indicated by the crystal structure (Tables 2–4) The natural ligands of class A GPCRs bind extracellularly to large soluble N-terminal ectodomains, i.e. the GPHRs. Consistent with the results of our analysis, it has been reported that the activity of these receptors can be modulated through allosteric ligands that bind within the 7-TM cavity in correspondence with the canonical orthosteric site of binding of class A GPCR ligands. This substantial topological conservation of the binding cavity of class A GPCR supports the applicability of molecular docking at GPCR homology models to computer-aided drug discovery [11].

As mentioned before, residues in EL2 are involved in ligand recognition and activation, and after including the EL2 domain to the class A subset, a pair of the EL2 positions had a significantly large degree to be classified as key positions. Additionally, two of the identified non-EL2 key positions are located at the C-terminal and N-terminal ends of TM4 and TM5, respectively, and can be regarded as the hinges of EL2. These two residues may play a role in the agonist-induced conformational changes of the loop that have been proposed to be required for receptor activation [21], [28].

Our algorithm finds a 7-TM binding cavity also for class C receptors, even though their natural ligands bind to their extracellular N-terminal domains. In agreement with our results, for several class C receptors have been reported allosteric modulators that bind to the 7-TM cavity [70]–[76]. The key positions identified here are all located within the two adjacent sites for two different classes of allosteric modulators of the Ca^2+^ recetor that have been proposed according to an experimentally supported *in silico* model [71] (Figure 7). Moreover, our analysis confirms that the high MI hub positions in class B receptors do not possess a well-defined 7-TM binding cavity for ligands that is shared by the entire class.

Our algorithm was able to highlight positions located in the exofacial ligand binding cavity, not those located within the 7-TM core or close to the intra-cellular region. Evolving from a common GPCR ancestor through the subsequent mutation of neighboring AA residues, GPCR binding cavities have diversified and acquired the ability of selectively recognizing specific ligands. Other biologically relevant GPCR residues – such as those important for structural integrity, activation, or G protein coupling – may be correlated with a limited number of partners, or may have remained too conserved during the evolution to be detected by our algorithm. We note that previous bioinformatic analyses involving GPCRs have uncovered residues located in the ligand-binding region as well as within the receptor core [15], [18], [19], [77]–[85], but whether all identified positions have the same statistical significance is not obvious.

The prediction of a cohort of functionally important, specificity determining, or coevolving residues, without involving MI, has also been addressed extensively [15], [18], [19], [57], [77], [78], [80]–[83], [83]–[117] and a few of these strategies were summarized by those previously cited and by Ortiz and colleagues [118] and Donald and Shakhnovich [36]. For class A GPCRs, investigators have used independent theoretical analyses [15], [18], [19], [77]–[85] involving the entire superfamily as well as select subfamilies from the class A superfamily. However, this is the first comprehensive investigation for correlated residues involving class B and C GPCRs.

The general significance of our results and previous findings that involve coevolving cohorts are supported in recent work from Ranganathan and colleagues [119]–[121]. They have demonstrated that maintaining the conservation pattern in a protein family, along with a small subset of coevolving residues, may enable the generation of low-homology sequences that fold and function. This work supports their finding that the number of key/critical interactions in a protein may be smaller than previously thought.

We propose that our algorithm could be used to identify positions as hubs of high MI. For an evolutionary diverse superfamily such positions can be involved in its structure/function such as ligand-binding, provided an accurate MSA is used as an input. If applied to families of proteins completely lacking three-dimensional information, our procedure could potentially lead to the identification of the residues lining the binding cavities solely from the alignment of homologous sequences provided those positions are not conserved. These theoretical predictions, in combination with experimental data, could be a great asset for the identification of ligand recognition sites and to the drug discovery process.

## Methods

### Data set

This work involves the published MSA of 358 non-olfactory human GPCRs due to Rognan and colleagues [15], with 287, 49 and 22 sequences for classes A, B, and C respectively. The list of sequences and their MSA is available at “human GPCR database” http://bioinfo-pharma.u-strasbg.fr/gpcrdb/gpcrdb_form.html web site. We also use the 249 sequences from class A for investigating 5 selected AA positions from EL2 in the proximity of TM3 (see MSA S1). To facilitate the comparison of AA residue positions among receptors, we identified the TM positions using the indexing scheme of Ballesteros and Weinstein [58]. In this scheme, the most conserved residue within a given TM is assigned a positional index X.50 (where X is the TM number), while the remaining residues are numbered relative to position 50. Similarly, here we assigned the positional index EL2.50 to the Cys in the second extracellular loop involved in the conserved disulfide bridge with TM3, and numbered the remaining EL2 residues relative to that position.

### MI estimation

The MI for an ordered set of position pairs *(j, k)*, which corresponds to two distinct positions within the MSA of the TM regions, is defined as
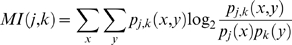
(1)where *p_j_(x)* is the estimated probability of AA *x* occurring at position *j*, and *p_j,k_(x,y)* is the joint probability of AAs *x* and *y* occurring at positions *j* and *k* respectively. The logarithm to the base 2 is an arbitrary choice. The sums are over the 20 naturally occurring AAs. Measured AA frequencies from the sequences in the data sets for each class are used to estimate the occurrence probabilities in Equation 1 and will be associated with uncertainty in the probability estimates due to random occurrences in a finite number of sequences. A null value of MI represents a sequence set in which all the positions in the alignment are completely independent, while a high MI value corresponds to high correlations between the position pairs. Since *MI(j,k) = MI(k,j)*, only *MI(j,k)* with *j<k* is computed.

We note that an equivalent definition of mutual information is in terms of the informational entropy:
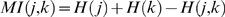
where 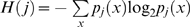 is the entropy and
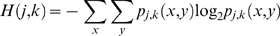
is the joint entropy.

### Finite size effect

We demonstrate that the estimated mutual information for a finite set of sequences with random AAs (i.e. with completely independent positions and hence theoretically zero MI) can have a nonzero MI that depends on the number sequences. Let *S* be the number of sequences in the set. Suppose that the true probability of the occurrence of AA *x* is *f_x_*, so *p(x)* = *f_y_*. The true joint probability of a pair of AAs is then *p(x,y) = f_x_ f_y_*. However, for small sets, if 1/*f_x_f_y_*<*S*, as in our case, then the most likely scenario is that the pair never appears and thus our estimate for *p(x,y)* is zero, which does not contribute to the *MI* computation. However, if a pair does appear then the lower bound of our estimate on its probability is 1/*S*. Hence, log[*p(x,y)/(p(x)p(y))*]∼log[1/*S*]. The sum in the *MI* will be dominated by terms to this order leading to a spurious estimate of the MI that is proportional to −log *S*.

The maximal MI for a set of sequences is obtained when *p(x,y) = p(x) = p(y)*. Thus:
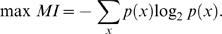
For *S*≫20 (the number of AAs), the above expression is limited by *p(x)*∼1/*20*, which in turn yields:
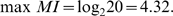
However, when S≤20, the nonzero lower bound on *p(x)* is 1/*S* and this in turn yields:
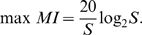



### MI graph construction

For a single GPCR there are 189 positions within the 7-TMs. We computed the MI for the 15,255 inter-TM position pairs. (For the class A subset involving EL2 there are 16,200 inter-TM position pairs). Using an ordered list of the top most MI pairs as edges, a MI graph was constructed. The graph consisted of vertices that corresponded to TM positions. We inserted an edge between two positions if the MI between those positions exceeded a MI threshold. This threshold was chosen such that the MI value would be significant with respect to a surrogate set of sequences (with the same number of sequences as the corresponding class) but with the TM residues randomized. The randomization was achieved by shuffling residues across sequences at a given alignment position as done by Mirny and Genfand [51]. This strategy preserved *p(x)* and *p(y)* but eliminated any correlation in the joint probability *p(x,y)*. As shown before, the estimated MI for a finite set of random sequences may be nonzero and will depend on the number of sequences in the set. We thus defined the significance threshold for MI in terms of the probability or P-value *P*_M_ of that MI value to occur in a surrogate set. Each *P*_M_ will yield a MI graph with *N* vertices and *M* edges.

### Key position identification

After constructing the MI graph, we identified the vertices in the MI graph with highly significant degree with respect to a null hypothesis of a randomly connected graph (Erdos-Renyi graph) with *N* vertices and *M* edges. The comparison was facilitated by constructing a distribution function for vertices with a given degree. We then established degree significance in terms of the probability or P-value *P*_D_ of a vertex of that degree to occur in a random graph as illustrated in Figure 5. For each M and *P*_D_, a ranked list of positions in terms of degree, which we call candidate positions, were obtained. The algorithm generating candidate positions is summarized in the flowchart in Figure 1.

As a criterion for identifying a unique and robust cohort of key positions, we insisted that the key positions be the candidate positions that are invariant to a leave-one-out analysis of the sequences (i.e. for *S* sequences, the analysis was repeated *S* times, each time leaving out one of the sequences) and over a range of *P*_D_ and *P*_M_. By invariant, we mean that there exists some ranking number *n* such that all positions with ranking higher than *n* retained a ranking higher than *n* (independent of order) over a range of *P*_D_, *P*_M_ and the leave-one-out analysis. We were not concerned if the rankings were permuted within the top *n* positions, they simply had to appear in the top *n* consistently. This invariant set defined the cohort of key positions. We note that our analysis did not require that the cohort of key positions reside in the same *clique* i.e. a mutually connected subgraph. We thus made the additional check of whether the key positions were directly connected by mutual information to other key positions and thus formed a clique.

### Additional tests

To further confirm our results, we performed an additional significance test for our high degree positions by testing significance with respect to a degree distribution that corrsesponds to a surrogate set of graphs generated directly from shuffled sequences.

We also selected positions using MI criteria proposed by Gloor et al. [38], [50]. They considered mutual information normalized by the joint entropy *MI*(*j,k*)/*H*(*j,k*) and set a threshold of Z-score>4.0 for accepting pairs of positions. Z-score is defined as (MI - <MI>)/σ_MI_, where <MI> is the mean and σ_MI_ is the standard deviation of the MI distribution. They then set a threshold of degree ≥ 3 for selecting positions. We performed this analysis for both normalized and un-normalized MI and for differing criteria for Z-score and degree.

To control against possible random or phylogenetic sources, we used the approach of Dunn, Wahl and Gloor [37] to reduce the background MI for every inter-TM position pair. The estimated average product correction (APC) for position pair (*j,k*) was obtained by taking the product of the average MI values across row *j* and column *k* illustrated in Figure 2 (using all position pairs). The relevant correction was then normalized by the overall average MI (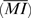), as
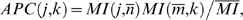
which is Equation (5) of reference [37]. 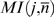 is the average MI in row *j*, and 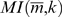 the average *MI* from column *k*. This unique correction was then subtracted from the raw MI value: *MI*(*j,k*). The leading corrected *MIp*(*j,k*) values were used to compute the MI graphs.

The significance and specificity of the identified key positions was evaluated using positional information from the crystal structure of β2-AR. From a total of 189 positions, the 18 ligand-binding positions of β2-AR are underlined in Table 2, column 2. For a specific threshold significance *P*_M_, the identified number of key positions represented the sum of true positives and false positives. The true positives were established by confirmation with the list of the underlined positions on Table 2. The false positive positions were those key positions not confirmed on the list of 18. The underlined positions from Table 2 that were not identified as key positions were classified as false negatives. Of the total 189 positions, those that were neither true positives nor false positives nor false negatives were true negatives. Sensitivity is the ratio of the number of true positives to the sum of true positives and false negatives. Specificity is the ratio of the number of false positives to the sum of false positives and true negatives. The sensitivity versus 1- specificity (ROC curve) was then computed.

### Additional Files

MSAof five contiguous EL2 residues of a subset of 249 class A receptors. The file (MSA S1.fst) is provided in FASTA format.

## Supporting Information

MSA S1 (0.00 MB DOC)
